# Linking the Metabolic Activity of Plastic-Degrading Fungi to Their Taxonomy and Evolution

**DOI:** 10.3390/jof11050378

**Published:** 2025-05-15

**Authors:** Anusha H. Ekanayaka, Namali T. De Silva, Entaj Tarafder, Xue-Mei Chen, Dong-Qin Dai, Steven L. Stephenson, Suhail Asad, Saowaluck Tibpromma, Samantha C. Karunarathana

**Affiliations:** 1 Center for Yunnan Plateau Biological Resources Protection and Utilization, College of Biology and Food Engineering, Qujing Normal University, Qujing 655011, China; hasinie88@gmail.com (A.H.E.); e3396023108@gmail.com (X.-M.C.); cicidaidongqin@gmail.com (D.-Q.D.); 2 Department of Urban Bioresources, Faculty of Urban and Aquatic Bioresources, University of Sri Jayewardenepura, Gangodawila, Nugegoda, Colombo 10250, Sri Lanka; 3 Department of Botany, Faculty of Applied Sciences, University of Sri Jayewardenepura, Gangodawila, Colombo 10250, Sri Lanka; namalidesilva97@gmail.com; 4 Department of Plant Pathology, College of Agriculture, Guizhou University, Guiyang 550025, China; entajmycology@gmail.com; 5 Center of Excellence in Microbial Diversity and Sustainable Utilization, Department of Biology, Faculty of Science, Chiang Mai University, Chiang Mai 50200, Thailand; 6 Department of Biological Sciences, University of Arkansas, Fayetteville, AR 72701, USA; slsteph@uark.edu; 7 School of Tea and Coffee, Pu’er University, Puer 665000, China; suhailasad@peu.edu.cn; 8 National Institute of Fundamental Studies (NIFS), Kandy 20000, Sri Lanka

**Keywords:** depolymerization, energy source, enzymes, metabolic by-products, mineralization

## Abstract

Plastic, a ubiquitous part of our daily lives, has become a global necessity, with annual production exceeding 300 million tons. However, the accumulation of synthetic polymers in our environment poses a pressing global challenge. To address this urgent issue, fungi have emerged as potential agents for plastic degradation. In our previous manuscript, ‘A Review of the Fungi That Degrade Plastic’, we explored the taxonomic placement of plastic-degrading fungi across three main phyla: Ascomycota, Basidiomycota, and Mucoromycota. In this review, we built upon that foundation and aimed to further explore the taxonomic relationships of these fungi in a comprehensive and detailed manner, leaving no stone unturned. Moreover, we linked metabolic activity and enzyme production of plastic-degrading fungi to their taxonomy and summarized a phylogenetic tree and a detailed table on enzyme production of plastic-degrading fungi presented here. Microbial enzymes are key players in polymer degradation, operating intra-cellularly and extra-cellularly. Fungi, one of the well-studied groups of microbes with respect to plastic degradation, are at the forefront of addressing the global issue of plastic accumulation. Their unique ability to hydrolyze synthetic plastic polymers and produce a wide range of specific enzymes is a testament to their potential. In this review, we gather and synthesize information concerning the metabolic pathways of fungi involved in the degradation of plastics. The manuscript explores the diverse range of specific enzymes that fungi can produce for plastic degradation and the major pathways of plastic metabolism. We provide a listing of 14 fungal enzymes (Esterase, Cutinase, Laccase, Peroxidases, Manganese peroxidase, Lignin peroxidase, Oxidoreductases, Urease, Protease, Lipase, Polyesterase, Dehydrogenase, Serine hydrolase, and PETase) involved in pathways for plastic degradation alongside the relevant fungi known to produce these enzymes. Furthermore, we integrate the fungi’s enzyme-producing capabilities with their taxonomy and phylogeny. Taxonomic and phylogenetic investigations have pinpointed three primary fungal classes (Eurotiomycetes, Sordariomycetes (Ascomycota), and Agaricomycetes (Basidiomycota)) as significant plastic degraders that produce the vital enzymes mentioned earlier. This paper provides a foundational resource for recognizing fungal involvement in the biodegradation of synthetic polymers. It will ultimately advance fungal biotechnology efforts to address the global issue of plastic accumulation in natural environments.

## 1. Introduction

Plastic, one of Earth’s most widely produced materials, is known for its durability and adaptability. Plastics can be broadly categorized into synthetic and biodegradable types. Synthetic plastics, derived from petrochemicals, and biodegradable plastics, manufactured from natural compounds, serve different purposes. Plastics can be broadly categorized based on their origin and degradability. In terms of origin, they can be classified as either synthetic or natural. Seven major types of synthetic plastics (polyethylene terephthalate (PET), high-density polyethylene (HDPE), polyvinyl chloride (PVC), low-density polyethylene (LDPE), polypropylene (PP), polystyrene (PS), and various other plastics) are used worldwide [1,2,3]. In terms of degradability, plastics can be further divided into those that are degradable by natural physical and biological factors and those that are non-degradable by natural forces, including biodegradable synthetic plastics.

The production and consumption of plastic are escalating annually, with global plastic production reaching 400.3 million metric tons in 2022 [4]. In 2018, Europe alone produced 61.8 million tons of plastic. However, only 9.4 million tons of post-consumer plastic waste were recycled during the same period [5,6,7]. This disparity underscores the inefficiencies in current waste management systems, leading to the accumulation of plastic waste in the environment. Approximately 109 million metric tons of plastic are estimated to have accumulated in rivers globally, while about eight million tons enter oceans annually. Plastics comprise approximately 80% of marine litter, with an estimated 30 million metric tons of plastic waste already accumulated. Projections suggest this figure could escalate to a staggering 150 million metric tons by the end of 2025 if we do not take significant interventions. Without such actions, a terrifying 12,000 million tons of plastic waste could reside in landfills or the natural environment by 2050, with annual accumulations reaching 339 million metric tons [8,9].

The environmental persistence of plastic wastes, due to their polymeric structure, high molecular weight, and hydrophobic nature, is a global issue. Certain synthetic plastics can persist for up to a millennium, and these properties also facilitate the breakdown of plastics into microplastics. These microplastics infiltrate ecosystems and food chains, posing uncertain but likely detrimental effects on biodiversity and human health. Consequently, plastic pollution has emerged as a critical global environmental challenge [2,10].

Biodegradation, a crucial process, has been proposed as a sustainable and efficient method for mitigating environmental plastic accumulation [2]. Elahi et al. [1] referred to biodegradation as any physical or chemical alteration in a material caused by microbial activity and harnesses microbial enzymes to facilitate the breakdown of synthetic polymers. The biological degradation process involves three stages: microbial attachment to the polymer surface, utilization of the polymer as a carbon source, and polymer degradation [11,12]. Microbial enzymes, which can act intra-cellularly or extra-cellularly, are central to this process. Given synthetic plastics’ large size and hydrophobic nature, extra-cellular enzymatic activity is particularly critical. This process, termed bio-fragmentation, reduces polymer chains into smaller, water-soluble monomers that are subsequently metabolized by microbial cells [13,14].

Among microorganisms, fungi have attracted significant attention for their capacity to degrade synthetic plastic polymers. Compared to bacteria, fungi are often more effective due to their ability to adhere to hydrophobic plastic surfaces and secrete robust extra-cellular enzymes capable of breaking down recalcitrant hydrocarbons [15,16,17]. This enzymatic capacity is closely linked to fungal genetic and phylogenetic characteristics, highlighting the potential for exciting future discoveries and evolutionary insights into their plastic-degrading capabilities. Such studies are essential for understanding and accelerating plastic biodegradation within natural ecosystems.

Our previous review thoroughly examined the taxonomy and phylogenetic relationships of major plastic-degrading fungal groups [2]. This paper extends that work by investigating the enzymatic mechanisms and metabolic pathways fungi use to degrade plastics, linking these processes to fungal taxonomy and phylogeny. Moreover, this study integrates novel findings from recent research [18,19,20] to provide a comprehensive understanding of fungal contributions to plastic degradation. This comprehensive understanding will keep you informed and identify avenues for future research.

## 2. Methodology

The information presented in this paper was derived from various sources, including scholarly papers, digital databases, and personal communications. Data on the enzymatic pathways of plastic-degrading fungi were collected primarily from Google Scholar, ResearchGate, PubMed, and Web of Science. An online literature search for different enzymes for different plastic types, the respective metabolic pathways, and factors affecting plastic biodegradation by fungi was carried out. The major keywords used during the search were plastic-degrading fungi, fungal metabolism, plastic degradation, plastic biodegradation, evolution of plastic-degrading fungi, metabolic pathways in fungi for plastic degradation, fungal enzymes for plastic degradation, plastic biodegradation, and fungal diversity. Single keywords or combinations were used during the search. Furthermore, we applied the following selection criteria: studies published in peer-reviewed journals within the last 10 years, articles directly related to metabolic pathways of plastic-degrading fungi, and research from reputable sources with a strong citation record. Additionally, we manually screened the abstracts of the top 100 most relevant results based on title matching and citation count.

The phylogenetic tree was obtained from Ekanayaka et al. [2]. Branches of the phylogram were collapsed to show generic levels. Summarised data on the enzyme production of fungi for plastic degradation were manually linked to the phylogenetic tree. Notes on major fungal enzymes and major fungal genera recorded for plastic degradation are provided. Discussions herein are based on the results of the literature review on enzymes, related metabolic pathways of fungi for plastic degradation, and the evolution of enzyme production of fungi to degrade plastics.

## 3. Results

### 3.1. Fungal Enzymes in Plastic Biodegradation

The biodegradation of plastic is affected by different factors, including the chemical structure of the polymer, environmental factors, microorganisms in the surrounding environment, and their capabilities. Plastics are long-chain polymers with a high molecular weight. Hence, the initial stage of biodegradation of plastics requires the structural weakening of those polymers [21]. The chemical factors of polymer structure, such as the molecular weight of monomers, hydrophobicity of the exposed area, melting temperature, crystallinity grade and structure, elasticity, specific chemical groups, and glass transition, are highly affected by its biodegradation rate [22,23]. Moreover, environmental factors, such as UV exposure, temperature, pH, and salinity, influence the biodegradation process [16]. Environmental factors can induce cracks and surface roughness in plastics, resulting in molecular alterations [24]. Primarily, these factors lead to plastics oxidation and introduction of carbonyl/carboxyl/hydroxyl groups to the polymer, diminishing its hydrophobicity and consequently enhancing its biodegradability [25,26,27]. Fungi take on a pivotal role when they proliferate on plastic surfaces, inducing further alterations in the physical properties of plastics through crack expansion and pore size enlargement. Moreover, microorganisms possess the ability to chemically degrade plastics by altering factors such as the pH, acidity, and salinity of their immediate microenvironment [28]. Furthermore, they break down the plastics into shorter chains through depolymerization. Fungal enzymes play a crucial role in this process. Fungal exoenzymes generate plastic intermediates with altered properties, enhancing their cellular assimilation [29]. Once these intermediates are formed and assimilated, intra-cellular enzymes come into play. Cells utilize them as carbon sources, breaking them down into water and carbon dioxide or methane to complete the mineralization process [28,30,31].

The primary categories of enzymes engaged in the biodegradation of plastics are hydrolases and oxidoreductases (Table 1). Fungal hydrolases such as lipases, carboxylesterases, cutinases, and proteases have the ability to alter the surface of the plastic, thereby enhancing its hydrophilicity [32]. Oxidoreductases, including laccases and peroxidases, participate in the breakdown of plastics into smaller molecules like oligomers, dimers, and monomers [33,34]. Because of the robust carbon–carbon (C–C) bonds they possess, plastic polymers like PE, PS, PP, and PVC require oxidation prior to undergoing the depolymerization process [35,36].

Plastics can be classified into three groups based on the extent of their biodegradability. These are (I) plastic polymers lacking pro-oxidants, (II) plastic polymers containing pro-oxidants, such as oxo-degradable polymers, and (III) biodegradable polymers. The first group may not be susceptible to enzymatic breakdown often because they lack functional groups or enzyme-sensitive ester bonds. Consequently, mechanical and thermal degradation are typically the primary modes of deterioration for this category. Conversely, the second and third groups often possess appropriate functional groups or enzyme-sensitive ester bonds, making them susceptible to photo-oxidation, thermo-oxidation, and enzymatic degradation [37].

Table 1 summarizes the major plastic-degrading enzymes recorded from fungi, and short notes on each enzyme are provided below.

**Table 1:** Major enzymes recorded from fungal genera/species for possible plastic degradation ability.

Enzyme	Fungi Recorded for the Production of Enzymes	Targeted Plastic Type	References
Esterases	*Aspergillus fumigatus* *A. flavus* *A. niger* *A. tubingensis* *Aureobasidium pullulans* *Cladosporium asperulatum* *C. montecillanum* *C. pseudocladosporioides* *C. tenuissimum* *Cladosporium* sp. *Curvularia senegalensis* *Embarria clematidis*	PUR, PVC, and PU	[33,38,39,40]
Cutinases	*Amycolatopsis mediterannei* *Aspergillus oryzae* *Fusarium solani* *Humicola insolens* *Moniliophthora roreri* *Thermobifida fusca* *Thermomyces lanuginosus*	PCL, PET, PES, PBS, and PBSA	[41,42,43,44,45,46]
Manganese peroxidases	*Alternaria alternate* *Aspergillus caespitosus* *A. terreus* *Bjerkandera adusta* *Eupenicillium hirayamae* *Paecilomyces variotii* *Phanerochaete chrysosporium* *Phialophora alba* *Polyporus brumalis* *Trametes versicolor*	Nylon, PE, and LDPE	[47,48,49,50,51]
Laccases	*Alternaria alternata* *Ascocoryne* sp. *Aspergillus caespitosus* *Aspergillus* sp. *A. terreus* *Clavariopsis aquatica* *Cochliobolus* *Eupenicillium hirayamae* *Lesiodiplodia theobromae* *Paecilomyces lilacinus* *P. variotii* *Paradendriphiella arenariae* *Phialophora alba* *Phoma* sp. *Pleurotus ostreatus* *Trametes versicolor* *Trichoderma harzianum*	Nylon, PE, LDPE, PP, DBP, DEP, and BPA	[29,51,52,53,54,55]
Peroxidases	*Alternaria alternata*, *Ascocoryne* sp. *Bjerkandera adusta* *Ceriporiopsis subvermispora* *Clavariopsis aquatica* *Dichomitus squalens* *Paradendriphiella arenariae* *Phanerochaete chrysosporium* *Phlebia radiata* *Phoma* sp. *Pleurotus* spp. *Trametes versicolor*	PE, DBP, and BPA	[21,54,55]
Lignin peroxidases	*Alternaria alternata* *Aspergillus caespitosus* *A. terreus* *Paecilomyces variotii* *Phanerochaete chrysosporium* *Phialophora alba*	PVC	[51,56]
Oxidoreductases	*Alternaria alternata* *Fusarium falciforme* *Lasiodiplodia theobromae* *Paecilomyces lilacinum* *Penicillium chrysogenum* *P. simplicissimum* *Phanerochaete chrysosporium* *Pleurotus ostreatus* *Purpureocillium lilacinum* *Trametes versicolor* *Trichoderma harzianum* *Zelererion maritimum*	Polyolefin (PE, PS, PP, and PVC)	[53,55,57,58,59,60,61,62]
Ureases	*Aspergillus fumigatus* *A. niger* *Cladosporium asperulatum* *C. montecillanum* *C. pseudocladosporioides* *C. tenuissimum* *Cladosporium* sp. *Embarria clematidis*	PU	[33,40]
Proteases	*Aspergillus fumigatus* *Cladosporium asperulatum* *C. montecillanum* *C. pseudocladosporioides* *C. tenuissimum* *Cladosporium* sp.	PU	[33]
Lipases	*Acremonium* sp. *Alternaria* sp. *Aspergillus flavus G10* *A. oryzae* *A. tubingensis* *Beauveria* spp. *Candida* spp. *Cryptococcus* spp. *Eremothecium* spp. *Fusarium solani* *Fusarium* sp. *Geotrichum* spp. *Humicola* spp. *Mucor* spp. *Ophiostoma* spp. *Penicillium citrinum* *Penicillium* spp. *Rhizomucor* spp. *Rhizopus* spp. *Thermomyces lanuginosus* *Trichoderma* spp.	PET, 3PET, PU, PBS, PBSA, PCL, and PBS	[41,44,45,63,64,65,66]
Polyesterases	*Beauveria brongniartii* *Papiliotrema laurentii* *Penicillium citrinum*	PU, PES, and PEA	[21,67]
Dehydrogenases	*Aspergillus fumigatus* *Papiliotrema laurentii*	PU, PES, and PEA	[67]
Serine hydrolases	*Pestalotiopsis microspora*	PUR	[68]
PETases	*Pestalotiopsis microspora*	PET	[21]

Note: PU; PUR, polyurethane; PVC, polyvinyl chloride; PCL, polycaprolactone; PET, polyethylene terephthalate; PES, polyethersulfone; PBS, polybutylene succinate; PBSA, poly(butylene succinate-co-butylene adipate); PE, polyethylene; LDPE, low-density polyethylene; PP, polypropylene; PS, polystyrene; DBP, dibutyl phthalate; BPA, bisphenol A; PEA, polyesteracetals.

### 3.2. Major Fungal Enzymes for Plastic Biodegradation

#### 3.2.1. Hydrolases (EC 3)

Hydrolases are one of the largest and most diverse enzyme families that catalyze the cleavage of chemical bonds by utilizing water as a hydroxyl group donor, resulting in the division of a large molecule into smaller, more biodegradable products [69,70,71,72,73]. Fungal hydrolases are widely known to degrade plastic components that contain ester, amide, or urethane linkages, such as PU, PET, PES, PBS, and PCL [28,36,41,42,63,72]. Hydrolases have hydrophobic clefts near their active sites, facilitating attachment to non-polar, hydrophobic plastic surfaces [74]. Thereby, they catalyze the hydrolytic cleavage of the plastic polymer chain, resulting in smaller monomers or dimers, which are absorbed by the fungi as a source of carbon and energy for their metabolism [75,76,77]. However, fungal hydrolases face challenges in efficient plastic degradation, due to their limited penetration ability to highly crystalline plastics and operational instability under industrial conditions [41,42,63].

##### Esterases (EC 3.1.1.x)

As hydrolases, esterases catalyze the hydrolysis of ester linkages in various polymeric substrates, leading to the breakdown of ester bonds and the formation of acid and alcohol-based molecules with polar functional groups [69,70]. In numerous studies of plastic biodegradation, cutinases and lipases, both forms of esterases, have been highlighted as exceptional hydrolases capable of enabling the fungal breakdown of polyester-based plastics such as PCL, PLA, PU, PRS, and PVC [33,38,39,40,41,42,63]. The mechanism of esterases involves a catalytic triad (Serine, Histidine, and Aspartate/Glutamate), which facilitates a nucleophilic attack on the carbonyl carbon of the ester bond, initiating chain scission that leads to molecular weight reduction and increased susceptibility to microbial attack [78,79]. Broad substrate specificity and potential for functioning under mild environmental conditions make them effective enzymes for plastic degradation [41,42,63]. Despite their potential, their activity on high-molecular-weight, highly crystalline polymers remains limited [41,42,63].

##### Cutinases (EC 3.1.1.74)

As extra-cellular serine esterases, cutinases possess the natural ability to hydrolyze cutin [71,72,73]. Mechanistically, cutinases operate by catalyzing the hydrolysis of ester linkages within plastics such as PCL, PET, PES, PBS, and PBSA, producing monomeric or oligomeric byproducts such as terephthalic acid, ethylene glycol, and lactic acid [41,42,43,44,45,46,74,80]. Kinetic studies reveal that cutinases exhibit substrate specificity determined by their active site architecture. For example, *Humicola insolens* cutinase demonstrates high catalytic efficiency towards PET due to its open, active site, which facilitates substrate accessibility [81].

##### Lipases (EC 3.1.1.3)

Lipases are extra-cellular triacylglycerol acyl hydrolase enzymes that catalyze the hydrolysis of ester bonds in triglycerols into glycerol and fatty acids. Due to the structural similarities between lipid esters and synthetic polyester linkages, lipases have demonstrated active degradation of PET, 3PET, PU, PBS, PBSA, PCL, and PBS by breaking down the lengthy carbon chains [41,44,45,63,64,65,66]. Lipases possess a hydrophobic lid domain that opens to expose their active site upon contact with hydrophobic, non-polar plastic surfaces, facilitating effective binding and interaction with the substrate [76,82,83].

##### Polyesterases (EC 3.1.1.x)

Polyesterases represent a family of hydrolytic esterases that hydrolyze polyesters by breaking down the ester bonds in the polyester backbone, effectively depolymerizing the large plastic molecules into smaller fragments [75,84,85]. These are mainly known for PET degradation, which often involves two types of polyesterases enzymes, PETase and MHETase, converting PET into monomers [86]. PETase enzymes begin the initial hydrolysis by directly attacking the PET and cleaving the ester bonds, releasing the intermediates like mono-2-hydroxyethyl terephthalate (MHET) and bis-(2-hydroxyethyl) terephthalate (BHET), which are then further degraded by MHETase enzymes into terephthalic acid (TPA) and ethylene glycol (EG) [86]. Other than PET, polyesterases are known to be effective in the degradation of PBT and PVC [84].

##### PETase Enzymes (EC 3.1.1.101)

PETases are a subclass of enzymes belonging to the polyesterases that catalyze the hydrolysis of PET by breaking its ester bonds and converting it into intermediates like MHET and BHET [86]. Initially, PETase enzymes were discovered from the novel bacterium Ideonella sakaiensis 201-F6, which utilized PET as a carbon source, minimizing the natural degradation period of plastics from many years to days [86,87,88]. Structurally, PETases possess an α/β hydrolase fold and relatively open active site, which facilitates better attachment of bulky PET chains [86]. The PET monomer intermediates are formed through nucleophilic attacks on the carbonyl carbon atoms of the ester bonds, and those intermediates, especially MHET, can be further cleaved by MHETase into TPA and EG, which is proven to work synergistically to degrade PET [86] completely.

##### Ureases (EC 3.5.1.5)

Ureases are nickel-dependent enzymes and members of the superfamily of amidohydrolases and phosphotriesterases that catalyze the conversion of urea into ammonia and carbon dioxide [89,90,91]. Ureases contribute to the degradation of some specific plastics, such as PUs containing urea and urethane linkages, effectively breaking down the polymer chain and making it more susceptible to further microbial degradation [33,40,89,92,93]. It has been found that several enzymes, like proteases and esterases, also synergistically incorporate with ureases for plastic degradation, where proteases hydrolyze urethane and amide bonds, and ureases attack the urea linkages [93]. However, the role of ureases is generally considered supportive rather than primary in plastic degradation, as their standalone degradation efficiency is low [40,93].

##### Proteases (EC 3.4.x)

Proteases are a large class of hydrolases that can cleave protein peptide bonds, resulting in peptides and amino acids [94,95]. In addition to their primary function, proteases such as cutinase-like enzymes (CLE) and metalloproteases like neprilysin (NEP) are found to be functional in plastic degradation, especially focused on PET and PUR [16,96]. They are known to follow different mechanisms in PET degradation, where CLEs use general acid/base mechanisms, including acylation and diacylation processes, and NEP utilizes either metal-bound hydroxide attachments or reverse protonation mechanisms [96]. The activity of proteases can be enhanced by plastic surface modifications that increase hydrophilicity and by utilizing them in combination with ureases and esterases [96].

##### Serine Hydrolases (EC 3)

Serine hydrolases are a distinct, large, and diverse family of enzymes that contain a conserved serine residue in their active site, which is pivotal in the hydrolysis of various bonds, including peptide, ester, or amide bonds in diverse substrates [78,79]. This diverse family is home to well-known enzymes like lipases, esterases, proteases, and amidases, which have been studied for their plastic degradation abilities targeting the PEs and PUs [78]. They participate in plastic degradation by modifying the plastic surface, increasing the hydrophilicity, and then breaking down the polymers through hydrolysis reactions [21,88]. As a collective group, they increase the hydrophilicity in plastic degradation by cleaving the polymer chains and then introducing polar functional groups such as carboxylic acids and hydroxyls, which are water-loving, making the plastic more susceptible to the attachment of microbes and degradation [75,76,77]. However, serine hydrolases face difficulties efficiently degrading high-crystallinity or crosslinked polymers [76,77].

#### 3.2.2. Oxidoreductases (EC 1)

Oxidoreductases are a large family of enzymes with 22 subdivisions, including important representatives such as laccases and peroxidases. The main function of oxidoreductases is catalyzing oxidation and reduction (redox) reactions by transferring an electron from the donor to an electron acceptor, incorporating many cofactors such as heme, flavin, and metal ions [97,98]. Among fungi, oxidoreductases are most common in white and brown-rot basidiomycetes involved in the oxidative degradation of lignocellulosic biomass [97]. Other than this, oxidoreductases also play a crucial role in plastic degradation through oxidation reactions, introducing oxygen atoms into the polymer structure, and therefore breaking down the strong carbon-carbon bonds in recalcitrant plastic polymer backbone, especially in PE, PVC, PP, and PS [53,55,57,58,59,60,61,62]. Also, introducing oxygen-containing groups like carbonyls and hydroxyls by oxidation makes the plastic more susceptible to hydrolysis, allowing synergic degradation with hydrolases [99]. Their effectiveness, however, can be limited by enzyme inactivation due to reactive intermediates or lack of suitable redox mediators in vitro [57,58,59,60,61,62].

##### Laccases (EC 1.10.3.2)

Laccases are copper-containing oxidases that can catalyze the oxidation of a wide range of substrates, especially phenolic and non-phenolic substrates [100,101,102]. Even though laccases do not directly participate in cleaving polymer chains, they can initiate the depolymerization of plastics by attacking carbon–carbon and carbon–heteroatom bonds, oxidizing the polymer chains, which reduces molecular weight and degradation of the plastic including Nylon, PE, LDPE, PP, DBP, DEP, and BPA [29,51,52,53,54,55]. Laccases catalyze the oxidation of plastics by reducing molecular oxygen to water, generating free radicals to increase the surface polarity and render it more susceptible to enzymatic or microbial degradation [21,76,103]. The substrate range and their efficiency on plastics can be enhanced by the addition of redox mediators such as ABTS (2,2′-azino-bis (3-ethylbenzothiazoline-6-sulphonic acid)) and HBT (1-hydroxybenzotriazole) along with the laccases [104]. However, their activity is often limited by poor enzyme–plastic interactions and possible inactivation under oxidative stress conditions [104].

##### Peroxidases (EC 1.11.1.x)

Peroxidases are oxidoreductase enzymes that catalyze the oxidation of organic and inorganic compounds utilizing hydrogen peroxide (H_2_O_2_) as the electron acceptor [105]. A large number of predominant peroxidases have been identified in species of fungi, including manganese peroxidases, lignin peroxidases, versatile peroxidases, and dye-decolorizing peroxidases, with production contingent upon the substrate on which the fungi are cultivated [106]. Key attributes of peroxidases include their lack of specificity and their capability to oxidize substrates with elevated redox potentials [106,107]. Such characteristics render these enzymes versatile in plastic degradation, oxidizing plastic surfaces and generating reactive oxygen species (ROS) that can lead to random scission of carbon–carbon and carbon–hydrogen bonds, particularly in PE, DBP, and BPA [21,54,55]. Some studies suggest that the plastic degradation by fungal peroxidases is closely connected to the mechanism of their lignin catabolism [108,109]. For example, laccase and peroxidase are actively involved in the depolymerization of hydrophobic plastics in an analogous manner to lignin hydrolysis.

##### Manganese Peroxidases (MnP) (EC 1.11.1.13)

Manganese peroxidases are heme-containing, H_2_O_2_-dependent glycoprotein peroxidases belonging to the family of oxidoreductases. The naturally occurring fungal MnP plays a pivotal role in depolymerizing plant lignin, hemicellulose, and cellulose [110,111,112]. In addition to their primary role, MnPs have properties of plastic degradation, mainly PE, Nylon, and LDPE, by oxidizing the plastic surfaces, thereby generating ROS to fragment long polymer chains [21,48]. MnPs catalyze the oxidation of manganese (II) (Mn^2+^) to manganese (III) (Mn^3+^), which acts as a mediator to oxidize substrates [110,111,113]. The released Mn^3+^ acts as an oxidant, attacking phenolic structures in the plastic, creating phenoxyl radicals, and these radicals undergo several reactions, leading to the breakdown of plastic into smaller, more biodegradable molecules [21,48]. Their main limitation lies in their dependence on specific cofactors (Mn^2+^, H_2_O_2_, and organic acids) and potential inactivation due to ROS accumulation [110,111].

##### Lignin Peroxidases (LiP) (EC 1.11.1.14)

Lignin peroxidases (LiPs) are heme-containing peroxidases that function as ligninolytic enzymes, facilitating the H_2_O_2_-dependent oxidative depolymerization of lignin [114,115]. LiPs demonstrate relatively broad substrate specificity, with a diverse range of catalyzed oxidative reactions, which are H_2_O_2_-dependent [116,117]. Possessing a high redox potential, lignin peroxidases exhibit promise in partially depolymerizing plastics with aromatic content, such as PE, PVC, and PCB, by generating ROS to initiate oxidative chain scission for enhancing the accessibility of plastic surfaces to other enzymes [51,56,88]. However, their instability in excess peroxide and limited production under non-ligninolytic conditions are significant challenges for environmental applications [51].

##### Dehydrogenases (EC1)

Dehydrogenases are a large group of enzymes that belong to a subset of oxidoreductases, and they catalyze oxidation–reduction reactions using coenzymes such as NAD+/NADP+ and flavins like FAD and FMN as electron acceptors [118,119]. Alcohol dehydrogenases can be considered the most studied type of fungal dehydrogenases, and they are involved in the reduction and oxidation of alcoholic compounds and, therefore, are mainly associated with ethanol metabolism and fermentative enzymes [120]. Dehydrogenases are not primary agents of polymer degradation but play auxiliary roles in plastic degradation pathways [121]. The initial oxidation and chain cleavage of PEA, PU, and PES are carried out by several oxidative enzymes like laccases, and the resulting smaller fragments are catabolized by dehydrogenases into monoesters, alcohols, aldehydes, and fatty acids [67,121]. These are then incorporated into metabolic pathways of microbial cells to facilitate the complete mineralization of these intermediates [121].

### 3.3. Evolution of Fungi to Produce Enzymes for Plastic Biodegradation: Enzymatic Adaptations and Taxonomic Involvement

Fungi possess remarkable enzymatic capabilities that enable them to degrade complex organic polymers. These abilities have evolved, equipping fungi to decompose recalcitrant materials such as lignocellulose [122]. Interestingly, certain fungi have also adapted the mechanisms used to degrade plant cell walls for the degradation of synthetic polymers like plastics [123]. Evolutionary processes, driven by environmental pressures and the presence of plastic waste in ecosystems, have promoted the emergence and enhancement of plastic-degrading capabilities among several fungal genera [124].

Many fungal genera, including *Aspergillus*, *Penicillium*, *Paecilomyces*, *Alternaria*, and *Phanerochaete*, have been reported to participate in plastic degradation. These genera exhibit varied ecological strategies, including saprophytic, pathogenic, and endophytic lifestyles, and they produce diverse extra-cellular enzymes such as laccases, manganese peroxidases (MnPs), lignin peroxidases (LiPs), esterases, and cutinases [125]. These enzymes, originally evolved for the degradation of plant-derived polymers, have been co-opted and adapted for plastic biodegradation [126].

For example, species of *Aspergillus* and *Penicillium*, commonly found in soil and decaying organic matter, have demonstrated the ability to degrade polyethylene (PE), polystyrene (PS), and polyethylene terephthalate (PET). Their high enzymatic activity, particularly of esterases and cutinases, contributes to the depolymerization of plastics into simpler compounds [127,128]. Similarly, *Paecilomyces variotii* has been reported to produce hydrolases and esterases capable of breaking down polyurethane (PU), indicating a possible evolutionary adaptation to synthetic substrates [129].

*Phanerochaete chrysosporium*, a model white-rot fungus, is well known for its lignin-degrading system and has also been studied for its potential to degrade plastics. Its peroxidase and laccase systems, critical in lignin degradation, promise to oxidize and break down synthetic polymers. The evolutionary expansion and diversification of its ligninolytic enzymes have possibly allowed this fungus to interact with xenobiotic compounds such as plastics [130].

Endophytic fungi like *Alternaria* have also demonstrated plastic-degrading potential, possibly due to their capacity to adapt to host-derived complex substrates and environmental stresses. In these fungi, enzyme promiscuity, where enzymes evolve to act on a range of structurally diverse substrates, may play a significant role in the initial steps of plastic degradation [21].

Furthermore, basidiomycetous fungi such as *Pleurotus ostreatus* are increasingly recognized for their contribution to plastic biodegradation, particularly through the secretion of oxidative enzymes. Studies suggest that these fungi can fragment polyethylene and polystyrene through the oxidative action of MnPs and laccases. Such abilities may be traced to their evolutionary adaptation to wood degradation and oxidative stress responses [131].

Collectively, the ability of these diverse fungal taxa to produce plastic-degrading enzymes is likely the result of evolutionary convergence, wherein different lineages have independently developed similar enzymatic strategies to exploit plastic as a potential carbon source [132]. The evolutionary history of these fungi suggests a flexible genomic and metabolic framework that enables adaptation to emerging ecological niches, including environments contaminated by plastic waste [21].

**Figure 1:**
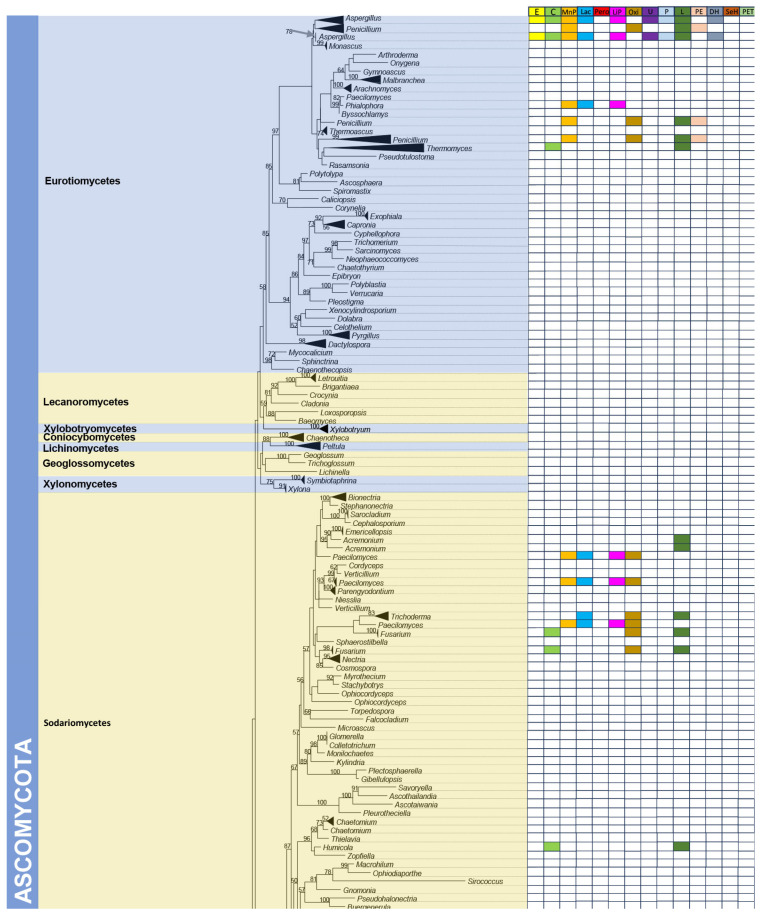

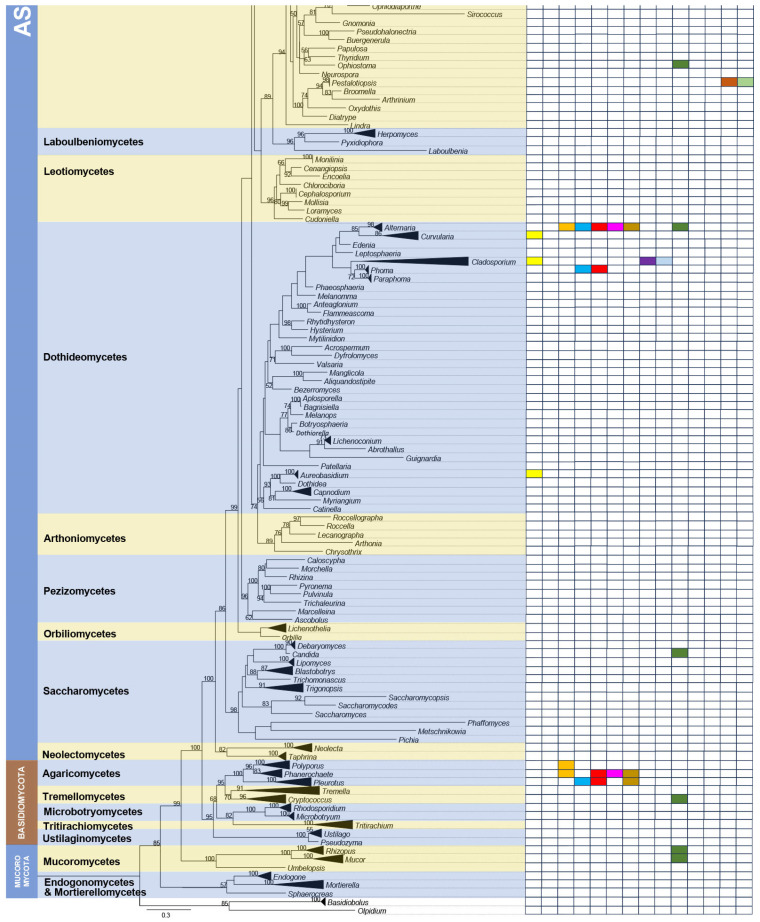
The phylogram was obtained from the data set provided by Ekanayaka et al. [2]. Branches are collapsed to show the genera of the kingdom Fungi. Fourteen columns on the right side of the figure include major fungal enzymes. The cells in each column are relevant to the fungal genera represented in the phylogram and are coloured accordingly when the fungal genera can produce enzymes. The literature summarized in Table 1 identifies each fungal genus’s capability to produce enzymes. Note: esterases, E; cutinases, C; lipases, L; laccases, Lac; peroxidases, Pero; manganese peroxidase, MnP; lignin peroxidase, LiP; oxidoreductases, Oxi; urease, U; protease, P; polyesterase, PE; dehydrogenase, DH; serine hydrolase, SeH; PETase, PET.

Various groups of fungi are engaged in the natural plastic degradation process. Based on data reported by Ekanayaka et al. [2], fungi capable of degrading plastics are found across three main phyla: Ascomycota, Basidiomycota, and Mucoromycota. The same phylogram published by Ekanayaka et al. [2] was used in the present study, and branches were collapsed to generic levels. Data on fungal enzyme production for plastic degradation were manually linked to the phylogram (Figure 1).

Based on the phylogenetic analysis, the fungal classes Eurotiomycetes, Sordariomycetes, and Agaricomycetes exhibit the strongest tendency toward plastic degradation. Within these classes, we observed six genera demonstrating a notable capacity to produce numerous enzymes crucial for plastic degradation. These are *Aspergillus*, *Penicillum*, *Paecilomyces*, *Alternaria*, *Phanerochaete*, and *Pleurotus*. Notes on each genus are provided below.

#### 3.3.1. *Aspergillus*

*Aspergillus* belongs to the family Aspergillaceae (Eurotiales, Eurotiomycetidae, and Eurotiomycetes). Index Fungorum summarizes over 1000 records for this genus [133].

The genus *Aspergillus* is able to produce diverse types of enzymes, such as amylase, lipase, protease, cellulase, pectinase, phytase, and β-glucanase, which are applied in a wide variety of industries, including food and dairy, textile, pulp and paper, leather, detergents, and waste management [134,135,136,137,138,139].

Based on our results, the genus shows a polyphyletic nature. *Aspergillus* spp. show promising applications in plastic degradation in producing a wide range of hydrolytic and oxidative enzymes, including esterases, cutinase, laccase, lipase, manganese peroxidase, lignin peroxidase, urease, protease, and dehydrogenases [2,21,83]. These enzymes are involved in depolymerizing PET, PES, PEA, PU, PBS, PBSA, PCL, and PBS, resulting in weight loss, surface cracking, erosion, and pore formation of these plastics [109,140].

#### 3.3.2. *Penicillium*

The genus *Penicillium* belongs to the family Aspergillaceae (Eurotiales, Eurotiomycetidae, and Eurotiomycetes). This is one of the largest genera within the kingdom of Fungi. Index Fungorum summarizes more than 1400 records for this genus [133].

The genus *Penicillium* is recorded to produce enzymes like xylanase, pectinase, amylase, peroxidase, laccase, and cellulase [141,142,143,144].

Based on our results, this genus also has a polyphyletic nature. One lineage of the genus grouped close to Aspergillus, while the other one grouped close to the genus *Thermomyces*. *Penicillium* seems to be one of the most successful genera for plastic degradation, displaying the capability of secreting a variety of enzymes, including esterases, cutinase, laccase, lipase, manganese peroxidase, lignin peroxidase, urease, protease, and dehydrogenase to biodegrade a panoply of plastic sources such as PUR, PVC, HDPE, LDPE, PHB, PLA, and epoxy resins through a process involving both oxidative and hydrolytic mechanisms [2,21,76,88,145]. It has been proved that *P. simplicissimum* can degrade polyethylene, without any additives or pre-treatments, solely using its laccases and manganese peroxidases, but the degradation showed high efficiency when the polyethylene is pre-treated with UV [60]. Also, *P. brevicompactum* was found to colonize and degrade e-waste PCB microplastics through chain scission and oxidation and achieved 75% mass loss within 28 days of incubation [146]. These findings reveal the potential of Penicillin in utilizing sustainable plastic waste management and other biotechnological applications.

#### 3.3.3. *Paecilomyces*

*Paecilomyces* belongs to the family Aspergillaceae (Eurotiales, Eurotiomycetidae, Eurotiomycetes, Pezizomycotina, and Ascomycota). Index Fungorum summarizes 151 records for this genus [133].

Most members of *Paecilomyces* have been identified as thermophiles and thermotolerants, and their known enzymes, such as chitinase, amylase, xylanase, hemicellulase, and pectinase, have drawn much attention from a wide variety of industries because of their thermotolerant properties [147,148,149].

Based on our results, the genus *Paecilomyces* can be separated into several lineages, thus showing its polyphyletic nature. One lineage was grouped within the Eurotiomycetes, while the others were grouped within the class Sordariomycetes. *Paecilomyces farinosus* and *Paecilomyces simplicissimum* have been studied extensively for their ability to degrade polythene substrates. They produce laccase, manganese peroxidase, lipase peroxidases, and oxidoreductases, which are common among other plastic-degrading fungi. They are most effective on biodegradable plastics such as PHB, Sky-Green, and PHBV films [2,150,151]. Segundo et al. [152] showed the possibility of *Paecilomyces* to reduce plastic waste and produce electrical energy in microbial fuel cells, displaying the potential of *Paecilomyces* in sustainable energy generation through their plastic degradation ability.

#### 3.3.4. *Alternaria*

The fungal genus *Alternaria* encompasses diverse ascomycetous fungi commonly found in soil, air, and decaying organic material. These organisms are widely recognized for their ecological roles as plant pathogens and saprophytes and their significance in industrial and medical mycology [153].

Emerging evidence indicates that species of *Alternaria* may contribute to the degradation of synthetic polymers. Recent studies have highlighted the ability of certain species of fungi to degrade synthetic polymers such as polyethylene (PE), polypropylene (PP), and polyurethane (PU) [154,155]. Species of *Alternaria*, known for their robust enzymatic machinery and adaptability to diverse substrates, have been implicated in this process. Enzymes such as cutinases, lipases, and esterases, commonly produced by *Alternaria*, are hypothesized to play a role in breaking down plastic polymers. However, the specific mechanisms and extent of plastic degradation by *Alternaria* remain largely unexplored [156]. Furthermore, a recent study examines the potential of species of *Alternaria* in plastic degradation. It critically examines the enzymatic pathways involved, the environmental conditions that facilitate such activity, and the limitations of current research [157].

The genus *Alternaria* belongs to the class Dothideomycetes, and in our phylogeny, it produces a stable lineage sister to *Curvularia*. Species of *Alternaria* produce lipase, laccase, peroxidases, manganese peroxidase, lipase peroxidase, and oxidoreductases.

#### 3.3.5. *Phanerochaete*

*Phanerochaete* belongs to the family Phanerochaetaceae (Polyporales, Incertae sedis, Agaricomycetes, Agaricomycotina, and Basidiomycota). Index Fungorum summarizes around 200 records for this genus [133].

*Phanerochaete* is considered an ecologically important genus that causes white-rot in both softwood and hardwood [158], and *P. chrysosporium* is regarded as the model organism for studies on wood and lignin degradation caused by white-rot species [159,160]. Also, these members are known to secrete extra-cellular non-specific enzyme complexes, including Class II peroxidases and glyoxal oxidases [161,162], which have attracted the attention of different industrial and biotechnological applications such as biofuel production, biopulping, bioleaching, and degradation of environmental pollutants, synthetic dyes, pesticides, and explosives [163,164,165,166,167,168].

*Phanerochaete* belongs to the Basidiomycota, and based on our results, this genus is phylogenetically close to the genus *Polyporus*. Species of *Phanerochaete* produce peroxidases, manganese peroxidase, lipase peroxidases, and oxidoreductases, which participate in depolymerization and degradation of PS, PLA, PE, and phenolic resin through different oxidation reactions [21,169,170,171]. Wu et al. [170] demonstrated that *Phanerochaete chrysosporium* could act as an efficient biodegrader for PLA and PS, where they achieved a mass loss of 34.35% and 19.71%, respectively, during 35 days of incubation.

#### 3.3.6. *Pleurotus*

*Pleurotus* belongs to the family Pleurotaceae (Agaricales, Agaricomycetidae, Agaricomycetes, Agaricomycotina, and Basidiomycota). Index Fungorum summarizes more than 700 records for this genus [133].

*Pleurotus* can secrete a range of enzymes, including peroxidases, laccases, cellulases, hemicellulases, and xylanases, which bring out the potential to be used in the bioconversion of agricultural wastes into value-added products, biodegradation of organic pollutants, toxins, industrial contaminants, and mycoremediation, especially concerning soil [172,173,174,175,176,177,178]. Their bioactive compounds also have potent anticancer, antibacterial, and antidiabetic properties, which have huge potential applications in the biomedical and pharmaceutical industries [179,180,181].

Based on our results, *Pleurotus* is a stable lineage sister to the genera *Polyporus* and *Phanerochaete*. *Pleurotus* produces a variety of enzymes such as laccases, peroxidases, and oxidoreductases, and these enzymes can break down complex plastic polymers such as PET, PE, PS, and PVC by oxidation [182,183]. The potential plastic degradation of *P. ostreatus* and *P. pulmonaris* has been displayed by several studies, both through visual and chemical changes of the plastic. The visual changes included discolouration, wrinkles on the surface, formation of holes, cracks, and crumbling, and for chemical changes, it was observed that the additions of degradation products such as hydrocarbons, carboxylic acids, alcohols, esters, and ketones [182,184,185].

## 4. Discussion

The growing concern regarding plastic accumulation in natural ecosystems has led to a growing interest in the biological degradation capabilities of microorganisms, particularly fungi, due to their capability in producing various enzymes and ecological adaptability [1,2]. Our review consolidates current knowledge on the fungal enzymes involved in plastic degradation and links these capabilities to fungal taxonomy and phylogeny. The findings highlight specific fungal clades, namely Eurotiomycetes, Sordariomycetes, and Agaricomycetes, as dominant groups possessing enzymatic machinery for plastic biodegradation.

Key fungal enzymes, cutinases, lipases, esterases, laccases, peroxidases, and oxidoreductases, play a significant role in the breakdown of synthetic polymers. Filamentous fungi mainly secrete these enzymes, and we identified some genera specific to this role: *Aspergillus*, *Penicillium*, *Paecilomyces*, *Alternaria*, *Phanerochaete*, and *Pleurotus*. These enzymes alter the polymer’s surface properties, later aiding depolymerization into smaller, metabolizable units. Furthermore, those enzymes increase the hydrophilicity of plastics and enable microbial assimilation [13,14]. Filamentous fungi, with their extensive hyphal networks and secretion of surface-active compounds like hydrophobins, are particularly effective in adhering to and degrading hydrophobic plastic materials [12].

By linking the enzyme-producing capability into fungal taxonomy and phylogeny in the present study, it was revealed that genera such as *Aspergillus*, *Penicillium*, *Phanerochaete*, and *Pleurotus* are strongly associated with the production of diverse enzyme types involved in plastic degradation. These genera in varied ecological niches have likely adapted to utilize synthetic polymers as supplementary carbon sources under nutrient-limited conditions [3,15]. Many of the enzymatic functions described originate from ancestral pathways associated with lignocellulose degradation, supporting the hypothesis that fungi have adapted these pathways to act on synthetic polymers. However, most enzyme evolution and activity assumptions remain speculative and require further empirical validation.

While these findings are promising, several constraints remain. Effective fungal plastic degradation is influenced by environmental conditions such as pH, temperature, and salinity, as well as the chemical complexity of the plastic itself [10,11]. Moreover, although laboratory studies demonstrate the potential of fungal enzymes in degrading plastics, field-level implementation is still limited by factors such as environmental variability, enzyme stability, and plastic additives. Hence, further research is necessary to improve enzyme stability and performance in diverse environments [13,14]. To enhance fungal degradation efficacy, future work should focus on isolating robust strains, characterizing novel enzymes, and applying molecular tools for genetic enhancement.

Many plastic-degrading enzymes share evolutionary origins with those involved in natural polymer degradation, such as lignin and cutin [15]. For instance, cutinases and laccases, enzymes originally evolved for plant biomass decomposition, have adapted to degrade synthetic polymers like PET and PE [12,13]. This supports the hypothesis of a metabolic shift in fungi driven by prolonged environmental exposure to plastic waste [2,6]. The specific genetic mutations and evolutionary pressures enabling plastic-degrading capabilities are poorly understood [8]. Therefore, to validate the enzymatic potential of fungi, future studies should prioritize functional characterizations, protein-level analyses, and advanced molecular tools beyond commonly used techniques like FTIR and general enzyme assays [2,12].

## 5. Conclusions

This paper consolidates existing information on the production of fungal enzymes for plastic degradation and explores their connection to taxonomy and phylogeny. It highlights the need for integrative approaches combining phylogeny, enzymology, and biotechnology.

Fungi, with their diverse array of enzymes tailored for decomposition, are the superior decomposers within natural ecosystems. Filamentous fungi exhibit a specialized degradation process, leveraging their exceptional metabolic versatility, secretion capabilities, and filamentous mycelial structure. Taxonomical and phylogenetic analyses have revealed that three primary fungal classes (the Eurotiomycetes, Sordariomycetes, and Agaricomycetes) significantly contribute to plastic degradation, producing relevant enzymes. Phylogenetic analyses reflect four phylogenetic clusters relevant to those fungi. In addition, a few other classes show a nascent capacity, indicating they are just beginning to develop this capability for plastic degradation. Our study has identified 14 key fungal enzymes crucial in plastic degradation.

Identifying novel fungal enzymes and improving their functionality through bioengineering could lead to scalable bioremediation strategies that address global plastic waste challenges.

## Data Availability

No new data were created or analyzed in this study.
